# Reappraising the TNM staging system for oral cavity squamous cell carcinoma: an age-related prognosis analysis

**DOI:** 10.1007/s00405-025-09385-x

**Published:** 2025-04-25

**Authors:** Amit Ritter, Eyal Yosefof, Nofar Edri, Noga Kurman, Gideon Bachar, Thomas Shpitzer, Aviram Mizrachi

**Affiliations:** 1 Department of Otolaryngology Head and Neck Surgery, Rabin Medical Center, Petah Tikva, Israel; 2 Faculty of Medicine, Tel Aviv University, Tel Aviv, Israel; 3 Davidoff Cancer Center, Rabin Medical Center, Petah Tikva, Israel

**Keywords:** Age factors, Carcinoma, Squamous cell, Neoplasm staging, Oral neoplasms, Prognosis

## Abstract

**Objective:**

Research on age and prognosis for oral cavity squamous cell carcinomas (OSCCs) has shown inconsistent results. We aimed to establish age as an independent prognostic factor and determine an age cutoff for staging in OSCC.

**Methods:**

Electronic records were reviewed for all OSCC patients treated between 2000 and 2020.

**Results:**

The study involved 250 patients, identifying a mortality cutoff age of 65 through Receiver Operating Characteristic curve analysis (sensitivity 77%, specificity 49%). Patients ≥ 65 had lower survival rates for early-stage (TNM I-II; 63.5% vs. 96%, *p* < 0.001) and advanced-stage (TNM III-IV; 37.5% vs. 62%, *p* = 0.011) diseases. A proposed age-based TNM staging system categorized ≥ 65 as Stage III/IV, with comparable survival rates confirmed in a revised analysis.

**Conclusion:**

The study identifies age, with a 65-year cutoff, as an independent prognostic factor in OSCC and highlights its role in improving current staging systems.

**Level of evidence:**

III.

## Introduction

There has been extensive research on the relationship between patient age and prognosis for oral cavity squamous cell carcinomas (OSCCs). So far, the results have been inconsistent. In some studies, younger patients had a better prognosis [1–3], while in other studies, the outcome was similar [4–7] or even worse [8–10]. Despite the varied results, the importance of patient age as a prognostic factor for OSCC cannot be overlooked.

In many studies on OSCC, a cutoff age of 40 years was used as a standard to distinguish between young and older adults, often with the aim of exploring differences in the etiology or biology of tumors across age groups [1–3, 6, 10, 11]. However, the primary objective of the present study was not to investigate etiological factors, but rather to evaluate age as a prognostic factor. Consequently, determining an age cutoff based on prognostic analysis is more appropriate for stratifying the OSCC patient population into risk groups. To that end, age-specific risk stratification based on prognostic data could offer a more reliable approach to differentiate between younger and older patients.

The American Joint Committee on Cancer (AJCC) staging system is widely utilized by clinicians and researchers to predict prognosis and guide treatment decisions for oral carcinoma [12]. The 8th edition of the AJCC staging system was significantly revised in 2017 by incorporating depth of invasion as a critical component of T staging, and extranodal extension was added to N staging in order to facilitate the assessment of patients with nodal disease more accurately [13]. Despite extensive research on the prognostic significance of the patient age for OSCC, age has not been considered when staging the disease. An example of age being incorporated as a factor influencing prognosis can be found in the AJCC classification system for differentiated thyroid cancer. As a result of the fact that younger patients usually have an excellent prognosis regardless of the course of their illness, the AJCC system assigned a different prognostic stage to those younger than 55 versus those aged 55 or older [14]. As with thyroid cancer, if it is determined that age is a significant indicator of prognosis for patients with OSCC, it could potentially be incorporated into the staging system.

We aimed to determine whether age should be considered an independent prognostic factor in patients with OSCC, and whether an age cutoff could be established for a possible age-based staging system.

## Methods

### Study design and subjects

The electronic records at Rabin Medical Center, a tertiary university-affiliated medical center, were reviewed for all patients who were treated for OSCC between 2000 and 2020. The study excluded patients with pathologically proven T0 disease (carcinoma in situ). The study protocol was approved by Rabin Medical Center’s Institutional Review Board.

### Data collection

The following data were collected from the patient files: demographics, risk factors for OSCC, clinical characteristics of the malignant disease, staging, treatment modalities, pathology reports, recurrence, and survival. The staging of OSCC was determined according to the 8th edition of the AJCC staging system for oral cavity cancer [13]. For cases treated prior to the 8th edition, staging was updated retrospectively. Adjuvant radiotherapy or chemoradiotherapy decisions were made by a multidisciplinary tumor board in accordance with the National Comprehensive Cancer Network (NCCN) guidelines relevant at the time of treatment. The follow-up period was defined as the time from surgical excision and diagnosis of malignancy to data collection for the study, with a minimum of 2 years or until patient death. Death was considered due to malignant disease in the following cases: complications related to distant metastases, direct local or regional complications of the malignant disease, surgical complications, and effects of chemoradiotherapy (CRT).

### Statistical analysis

All statistical analyses were performed with SPSS v.23.0 (IBM Corp., Armonk, NY, USA). Associations between nominal variables were examined using the χ2 test and Fisher’s exact test. Associations between continuous and quantitative variables were examined using Student’s t-test for normally distributed data, and Mann-Whitney U test as a nonparametric alternative. A Receiver Operating Characteristic (ROC) curve analysis was conducted to determine the cutoff age for survival prediction. Survival analysis was performed using the Kaplan–Meier method; the log-rank test was used to compare survival between groups. A two-sided p-value of < 0.05 was considered statistically significant for all analyses.

## Results

### Analysis of survival as a function of age

**Fig. 1:**
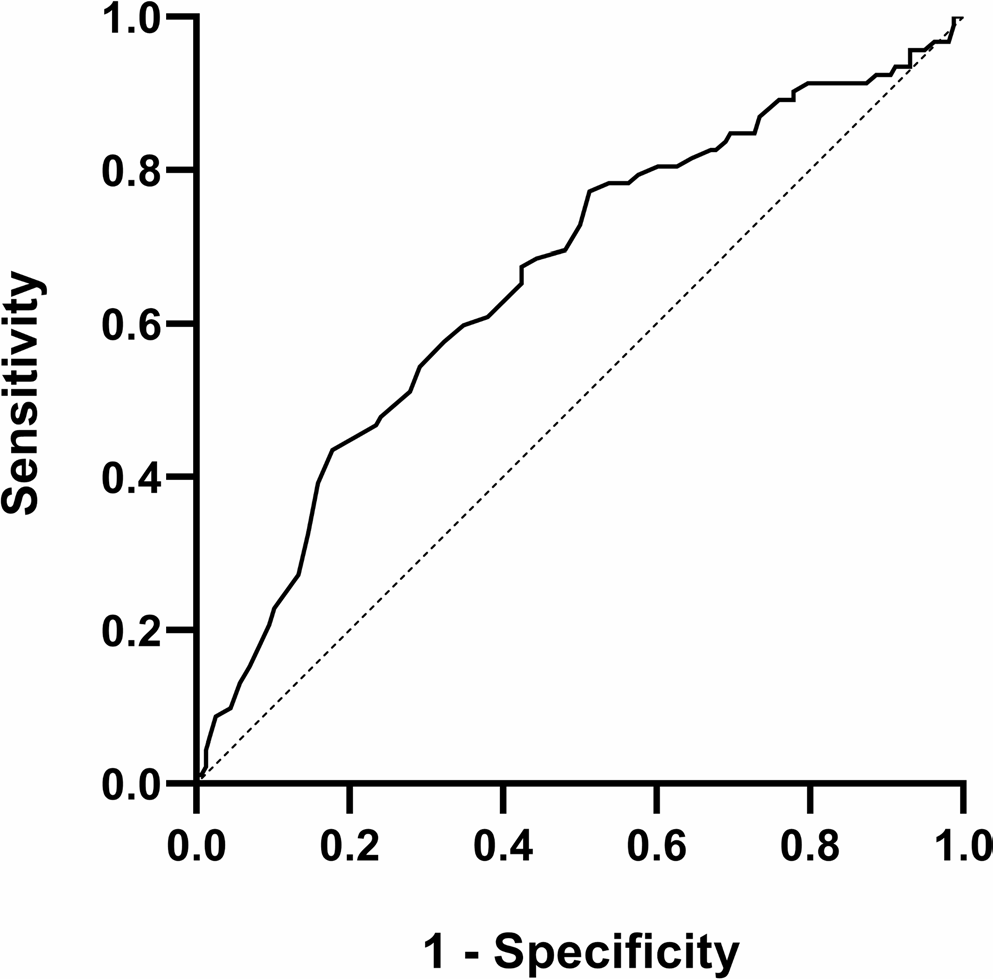
Receiver operating characteristic (ROC) curve analysis of age for the prediction of mortality in patients with oral cavity squamous cell carcinoma

There were 250 patients included in the study with a median age of 70 (range, 17 to 94 years). Twelve percent of the patients were younger than 40 at the time of diagnosis, and 22% were younger than 50. Patients were followed for a median of 48.5 months (range, 10 days to 258 months). At the conclusion of the follow-up period, 92 patients (37%) died of OSCC. Based on a ROC curve analysis of age for the prediction of mortality during follow-up, an area under the curve (AUC) with potential prognostic value was identified (AUC = 0.658; 95% confidence interval: 0.586–0.729) (Fig. 1). A 65-year cutoff age was determined to predict mortality based on Youden’s index (sensitivity 77%, specificity 49%). The cohort was divided into two groups based on the results.

### Comparison of baseline characteristics between age groups

A comparison of the baseline characteristics of the two age groups is illustrated in Table 1. The patient population below the age of 65 predominantly consisted of males, whereas the older patient group was predominantly composed of females (60% versus 42%, *p* = 0.005). As anticipated, younger patients demonstrated higher ECOG performance scores when compared to their older counterparts (*p* < *0.001*). Nevertheless, they also exhibited increased rates of smoking (48% versus 27.5%, *p* = 0.001). Mobile tongue was the most common subsite associated with cancer in both groups. Other subsites, including the buccal mucosa (12% versus 2%) and alveolar ridge (9% versus 4%), were more frequently involved in patients aged 65 and older (*p* = *0.004*). There was no significant difference in the rate of bony involvement and need for mandibulectomy or maxillectomy between the groups (25.5% versus 16%, *p* = 0.059). Similarly, there was no difference in the rate of free flap reconstruction (39% versus 32%, *p* = 0.289). Despite the fact that there was no significant difference in the clinical involvement of cervical lymph nodes between the two groups, patients in the advanced-age group underwent significantly fewer elective neck dissections for clinical N0 disease (60.5% versus 95.5%; *p* = 0.001). Comparing the TNM staging of patients by age group revealed no significant differences. Adjuvant radiation therapy rates were also comparable. Despite the expectation of lower rates of adjuvant chemotherapy in elderly patients, there were no significant differences between the two groups (20.5% compared to 30.5%; *p* = 0.066).

**Table 1:** Clinical characteristics of patients with oral squamous cell carcinoma

Characteristic	Age < 65 years (no. of patients = 98)	Age ≥ 65 years (no. of patients = 152)	*p*-value
Male gender, n (%)	59 (60)	64 (42)	0.005
Median age, year (range)	49 (17–64)	77.5 (65–94)	< 0.001
Risk factors, n (%)			
Smoking	47 (48)	42 (27.5)	0.001
Ethylism	6 (6)	4 (2.5)	0.198
Lichen planus	7 (7)	27 (17.5)	0.017
ECOG performance scale, n (%)			< 0.001
Grade 0	16 (16.5)	1 (0.5)	
Grade 1	11 (11)	26 (17)	
Grade 2	39 (44)	67 (44)	
Grade ≥ 3	4 (4)	14 (9)	
Tumor subsite, n (%)			0.004
Tongue	86 (87.5)	98 (64.5)	
Buccal mucosa	2 (2)	18 (12)	
Alveolar ridge	4 (4)	14 (9)	
Other sites	6 (6)	22 (14.5)	
Clinical N-positive, n (%)	31 (31.5)	33 (22)	0.079
Elective neck dissection, n (% of N-negative)	64 (95.5)	72 (60.5)	< 0.001
Bony involvement^a^, n (%)	25 (25.5)	24 (16)	0.059
Free flap reconstruction, n (%)	38 (39)	49 (32)	0.289
Pathologic TNM stage, n (%)			0.261
Stage I	33 (33.5)	56 (37)	
Stage II	15 (15.5)	35 (23)	
Stage III	22 (22.5)	23 (15)	
Stage IV	28 (28.5)	38 (25)	
Pathologic features, n (%)			
Poorly differentiated	14 (14.5)	40 (26.5)	0.506
Positive margins	7 (7)	15 (10)	0.662
Perineural invasion	18 (18.5)	27 (18)	0.523
Extranodal extension	7 (7)	14 (9)	0.785
Adjuvant treatment, n (%)			
Radiotherapy	48 (49)	72 (47.5)	0.803
Chemotherapy	30 (30.5)	31 (20.5)	0.066

^a^ Bony involvement: marginal mandibulectomy, segmental mandibulectomy, or maxillectomy

### Disease outcomes

**Fig. 2:**
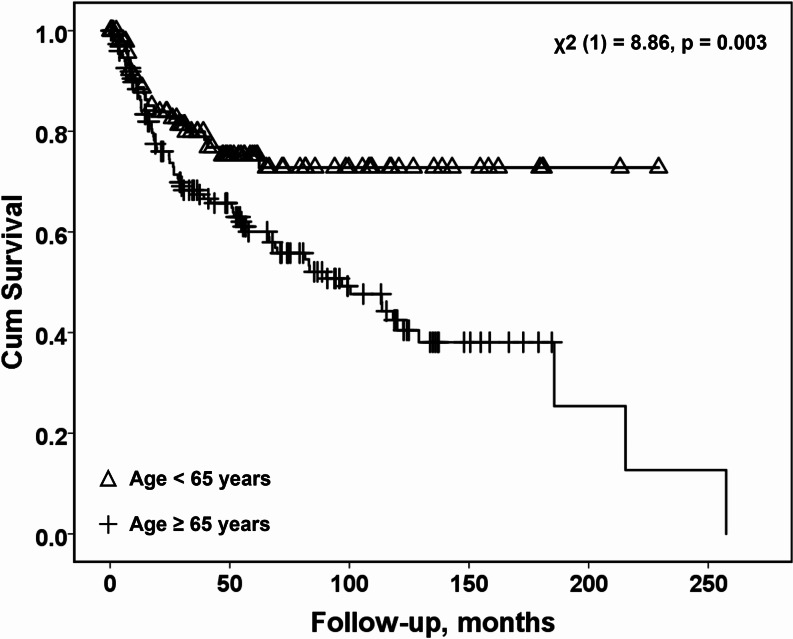
Survival analysis of patients with oral cavity squamous cell carcinoma: under 65 years vs. 65 years and older

Disease-specific survival (DSS) and disease-free survival (DFS) rates differed significantly between age groups. Eight patients under 65 years old were diagnosed with disease recurrences (8%), and 21 patients died of disease (21.5%) within a median period of 14 months (range, 3 months to 5 years). The advanced-age group of patients aged 65 years and older had 29 cases of recurrence (19%), and 71 cases of death from disease (46.5%) within a median of 24.5 months (range, 2 months to 21.5 years). A significant increase in disease-specific mortality was observed in patients ≥ 65 years (OR = 3.22, 95% CI: 1.82, 5.71; *p* < 0.001). Accordingly, survival analysis using Kaplan-Meier with log-rank test revealed significantly lower DSS in patients ≥ 65 years (χ2 (1) = 8.86, *p* = 0.003) (Fig. 2). The potential confounding variables, which were significantly different across age groups (see Table 1), were examined with univariate analysis, and their associations were examined with multivariate logistic regression (Table 2). Patients’ age, with a suggested cutoff value of 65 years, was the only variable independently related to disease-specific mortality (OR = 2.99, 95% CI: 1.12, 8.00; *p* = 0.03).

**Table 2:** Univariate and multivariate analyses of risk factors for disease-specific mortality

Risk factor	No. of patients	No. of events (%)	Unadjusted odds ratio (95% CI)	*p*-value	Adjusted odds ratio (95% CI)	*p*-value
Age group						
< 65 years	98	21 (21.5%)	Reference	< 0.001	Reference	0.030
≥ 65 years	152	71 (46.5%)	3.22 (1.82, 5.71)		2.99 (1.12, 8.00)	
Gender						
Male	123	40 (32.5%)	Reference	0.167	Reference	0.381
Female	127	52 (41%)	1.44 (0.86, 2.42)		1.44 (0.64, 3.28)	
Smoking						
No	161	63 (39%)	Reference	0.304	Reference	0.888
Yes	89	29 (32.5%)	0.75 (0.44, 1.30)		0.94 (0.40, 2.23)	
ECOG performance scale						
---	---	---	---	---	1.46 (0.82, 2.63)	0.202
Lichen planus						
No	216	76 (35%)	Reference	0.182	Reference	0.469
Yes	34	16 (47%)	1.64 (0.79, 3.39)		1.45 (0.53, 3.97)	
Tumor subsite						
Tongue	184	58 (31.5%)	Reference	0.004	Reference	0.282
Other	66	34 (51.5%)	2.31 (1.3, 4.10)		1.68 (0.65, 4.34)	
Elective neck dissection (% of N-negative)						
No	50	16 (32%)	Reference	0.815	Reference	0.202
Yes	136	46 (34%)	0.92 (0.46, 1.84)		1.59 (0.38, 1.23)	

**Fig. 3:**
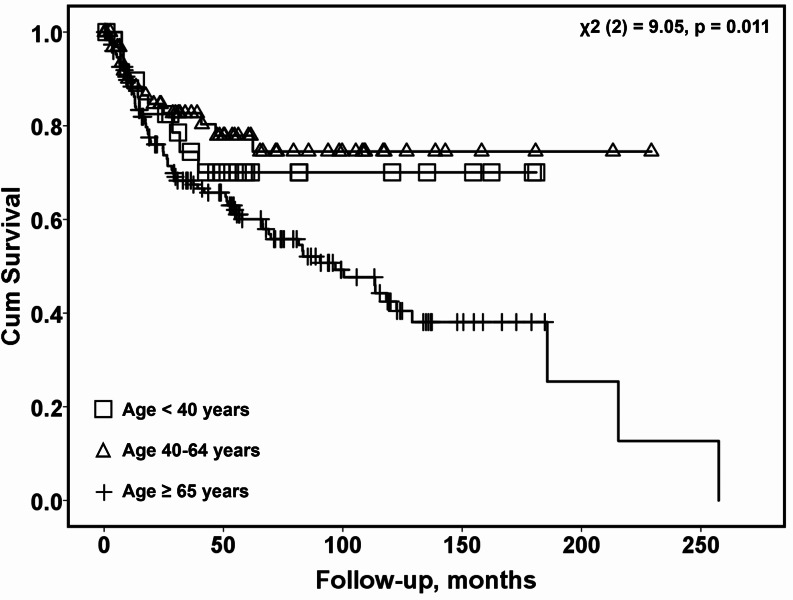
Survival analysis of different age groups with oral cavity squamous cell carcinoma

An additional survival analysis was conducted to address different age cutoffs suggested in previous publications. The cohort was stratified into three age groups: under 40 years of age, 40 to 65 years of age, and 65 years or older. There was no association between age under 40 and mortality (*p* = *0.22*), while age between 40 and 64 was associated with an improved disease-related survival rate (*p* < *0.001*). An increase in disease-related mortality was observed only among patients 65 years and older (*p* < *0.001*). The Kaplan-Meier survival curves for the three age groups are shown in Fig. 3.

### Re-evaluation of TNM staging

In both age groups, patients were stratified into four TNM stages (I to IV), and survival analyses were conducted for each stage (Table 3). Patients with early-stage disease (TNM I-II) had a lower survival rate if they were ≥ 65 years of age compared to those who were younger (63.5% versus 96%; *p* < 0.001). The survival rate for patients with advanced-stage disease (TNM III-IV) also differed significantly by age group (37.5% versus 62%; *p* = 0.011). A new age-related TNM staging system was proposed based on these findings to match the survival of advanced-age patients with their younger counterparts, and thus to improve survival prediction (Table 4). Patients aged 65 years or older were considered to have Stage III or Stage IV diseases: it was determined that T1-T2N0M0 diseases would be reclassified as Stage III, while N1 diseases would be reclassified as Stage IV. A revised comparative analysis based on the proposed classification revealed similar survival rates for both age groups (Table 3, right columns).

**Table 3:** Stage and prognosis according to TNM classification

TNM stage	*p*-value	Survival (%) Age ≥ 65 years	Survival (%) Age < 65 years	Survival (%) Age ≥ 65 re-classified	*p*-value
Stage I	< 0.001	35/56 (62.5%)	32/33 (97%)		
Stage II	0.076	23/35 (65.5%)	14/15 (93.5%)		
Early stage (I-II)	< 0.001	58/91 (63.5%)	46/48 (96%)		
Stage III	0.167	11/23 (48%)	15/22 (68%)	61/100 (61%)	0.529
Stage IV	0.038	12/38 (31.5%)	16/28 (57%)	20/52 (38.5%)	0.109
Advanced stage (III-IV)	0.011	23/61 (37.5%)	31/50 (62%)	81/152 (53%)	0.282

**Table 4:** A new age-related TNM classification of oral carcinoma

Under 65 years	T	*N*	M	65 years and older
Stage I	T1	N0	M0	Stage III
Stage II	T2	N0	M0	
Stage III	T3	N0	M0	
	T1/T2/T3	N1	M0	Stage IV
Stage IV	T4a/T4b	Any N	M0	
	Any T	N2/N3	M0	
	Any T	Any N	M1	

## Discussion

The last three decades have seen a rise in the global incidence of OSCC, particularly among people under the age of 50 [15]. Previous studies have examined the clinical course and prognosis of young patients with OSCC. Nonetheless, the definition of young age and its relationship to prognosis for patients with oral carcinoma have long been topics of discussion in the literature.

Many studies on oral cancer in the young population have used cutoffs ranging from 30 to 45 years of age [1–3, 5, 6, 10, 11]. This may be attributed to accepted definitions of ‘young adults’, as well as the prevalence of oral cancer at an early age. Approximately 5–10% of oral cancer patients are diagnosed before they reach the age of 40–50 [15, 16], and a growing body of evidence suggests traditional risk factors do not have a significant impact on cancer at these ages [11, 17, 18]. However, in order to determine the role of age as a prognostic factor for oral cancer, it must be examined independently of the classical definition of young adults and the low incidence of cancer in younger patients. In the present study, 12% of patients were diagnosed before the age of 40. Contrary to previous studies, this study utilized ROC curve analysis to determine the cutoff age for predicting mortality. A 65-year cutoff age was identified with an estimated 77% true positive rate and a prognostic value of > 0.65, which is likely to increase with a larger cohort. Patients were subsequently divided into two groups according to the 65-year cutoff.

Since patients were not matched for baseline characteristics, several variables differed significantly between the groups. Similar to previous studies, the oral tongue was the most common subsite for cancer development, with higher rates at younger ages (87.5% versus 64.5%). 19–21 Clinical TNM staging and the rate of neck metastases did not differ significantly between the two groups. The rate of elective neck dissection for N-negative disease, however, was significantly lower in the group of patients aged 65 and older. A possible explanation for this can be found in the adoption of an approach that recommends avoiding neck dissection complications in elderly patients who have early-stage cancer, as has already been suggested in previous publications [22, 23]. 

The survival outcomes of patients ≥ 65 years of age were less favorable even after accounting for potential confounders such as performance status, tumor location and elective neck dissection. In fact, patients of advanced age were three times more likely to die from OSCC than those of a younger age group. To date, the literature on the survival of young patients with oral cavity carcinoma has been inconclusive. In a recent population-based study by Oliver et al., the authors reported improved survival rates in patients younger than 40 years [2]. Another population-based study by Mukdad et al. demonstrated a similar trend with improved overall and disease-specific survival among patients younger than 40 years of age [3]. In contrast, other studies reported poor prognosis in young patients with oral carcinoma. According to Sun et al., patients younger than 40 years of age were more likely to experience recurrence of disease [9], while Jeon and colleagues reported 5-year survival rates of 42% in patients younger than 40 years, compared to 70% in older patients [10]. Some possible explanations for the conflicting reports in the literature include the use of arbitrary age cutoffs, the use of univariate models which are prone to confounding bias, or the use of different outcome variables to determine prognosis.

Having identified age as an independent predictor of prognosis, the survival rates of patients at each TNM stage were compared between the two age groups. We found that the survival rate of patients 65 years and older with early-stage cancer was similar to that of younger patients with advanced-stage cancer (62% and 63.5%, respectively). Expectedly, individuals aged 65 and older with advanced-stage cancer had lower survival rates than their younger counterparts (37.5% versus 62%). Although these findings highlight the potential importance of age as a prognostic factor, further research is required to clarify its exact role in disease staging and treatment decision-making. Currently, the TNM staging system does not account for age, and it remains unclear whether incorporating age into the system would provide a more accurate prediction model. In light of these findings, we explored a conceptual model that takes age into account when assessing prognosis, similar to the AJCC staging system for thyroid cancer [14]. This approach may help refine risk stratification, potentially informing future modifications to staging systems or even facilitating the development of a tool that incorporates age alongside other factors to guide individualized predictions. However, such an approach would require extensive validation before any clinical implementation could be considered.

This study indicates that age significantly impacts prognosis; however, it remains unclear whether age should also be considered as an independent factor when determining treatment options. According to the NCCN guidelines, adjuvant radiotherapy should be considered for patients with advanced-stage cancers, including T3-T4 and N2-N3 pathologies [24]. Adjuvant therapy is also recommended for patients with early-stage cancers with pathologically adverse features, especially when the surgical margins are close or involved. Yet, age has not been considered an independent determinant of the need for adjuvant therapy. Radiation therapy is well established in the elderly population, even in individuals over the age of 85 [25]. Contrary to this, the use of concomitant chemotherapy in patients over the age of 70 years should be used cautiously [26]. Considering the generally poorer prognosis of older patients with OSCC, further research may be warranted to assess whether adjuvant therapy should be routinely considered in this age group. However, until stronger evidence supports such a strategy, age should be viewed as a factor that supplements, rather than replaces, existing decision-making criteria.

The present study had several limitations, mainly due to its retrospective design and a relatively small sample size. Nevertheless, the results of the ROC curve analysis demonstrated potential prognostic value for age-grouping, supporting the conclusion that larger cohort studies will increase the predictive value of age on mortality. Due to the retrospective nature of the study, detailed comorbidity data were not consistently available. While acknowledging the potential impact of unmeasured comorbidities, we focused on disease-specific survival, which is less dependent on comorbidities than overall survival. Additionally, ECOG performance status data were available as a surrogate for overall health. The study was further limited by the fact that there was no matching of baseline characteristics between age groups. To overcome this limitation, potential confounding variables were identified, and a logistic regression was conducted to control for confounding bias. Furthermore, there is a possibility that the study results may have been skewed toward a lower percentage of Stage IV patients due to insufficient data collected on non-surgical patients. However, it is anticipated that any such bias would be minimal, as the majority of patients with OSCC are eligible for surgery irrespective of their age. Finally, the nature and biology of oral cancer may vary across different geographical regions. Since this study was based on single-center data without external validation, its conclusions should be interpreted with caution and considered specific to oral cancer in this region.

## Conclusions

Using a cutoff of 65 years to distinguish between young and old adults, this study suggests that age may be an important independent prognostic factor in patients with OSCC. While the findings indicate that age could supplement existing staging systems, further large-scale studies with external validation are required before considering any formal modifications.
